# In reply to “Anti-alpha-enolase antibodies in membranous nephropathy: isotype matters”

**DOI:** 10.1007/s10157-016-1291-7

**Published:** 2016-06-15

**Authors:** Hirokazu Imai, Yukihiro Kimura

**Affiliations:** Division of Nephrology and Rheumatology, Department of Internal Medicine, Aichi Medical University School of Medicine, Nagakute, Aichi 480-1195 Japan

*To the Editor*

We thank Corrado Murtas et al. for their attention to our paper that demonstrated the importance of anti-α enolase (AENO) antibodies in primary and secondary membranous nephropathy (MN). We emphasized the presence of anti-AENO IgG1 and IgG4 subclasses of antibodies in primary MN, and IgG1 and IgG3 subclasses in secondary MN, including lupus nephritis (type V) and bucillamine-related MN [1].

Murtas et al. reported the coexistence of different circulating anti-podocyte antibodies in MN [2]. They emphasized anti-AENO IgG4 antibodies. In the present study, our data were consistent with theirs for primary MN. However, for secondary MN, depending on autoimmune type, IgG1 and IgG3 subclasses were predominant. They also commented a high prevalence of anti-AENO IgG2 antibodies in lupus nephritis [3]. However, we did not detect anti-AENO IgG2 antibodies. We compared the dot blot methods for anti-AENO antibody between our study and Bruschi’s study (Table 1). Major differences are blocking reagents for nonspecific binding (skim milk versus BSA), and the dilution titers of patients’ sera (1:200 versus 1:50) and antibody for human IgG subclasses (1:500 versus 1:3000). Further study will be needed to confirm the best method for detecting anti-AENO antibody.

**Table 1:** A comparison on dot blot methods for anti-α enolase (AENO) antibody

	Kimura et al. [1]	Bruschi et al. [3]
Patients	25 patients with primary MN, and 13 patients with lupus nephritis (V)	20 patients with lupus nephritis (II, III, IV, V)
Nitrocellulose membranes	Nitrocellulose membranes (GE Healthcare, UK)	Bio-Dot apparatus (Bio-Rad, Hercules, CA, USA)
Antigen: α-enolase	Recombinant: home made	Recombinant: Abnova Corporation (Taipei, Taiwan)
Antigen amounts	10 μg/ml of enolase in Tween-PBS, for 60 min at room temperature	100 ng of enolase in TBS, for 4 h at room temperature, and then at 4 °C overnight
Blocking of nonspecific binding	5 % (w/v) skim milk (BD Difco, USA)	5 % (w/v) albumin in Tween-PBS
Patients’ sera	Diluted 1:200 in Tween-PBS at room temperature overnight	Diluted 1:50 in TBS-T (TBS-T 0.05 % v/v, 1 % w/v albumin) for 6 h at room temperature, and then at 4 °C overnight
Antibody	Peroxidase–conjugated mouse monoclonal antibodies to human IgG1, IgG2, IgG3, IgG4 (Invitrogen, USA) at diluted 1:500 for 2 h at room temperature	HPR–anti human IgG1, IgG2, IgG3, IgG4 (Invitrogen, USA) at diluted 1:3000 for 4 h at room temperature
Results	18 of 25 sera (IgG1 and IgG4, 11 patients; IgG4 alone, 6 patients; IgG1 alone, 1 patient) showed anti-AENO antibody in primary MN, and positive IgG1 and IgG3 anti-AENO antibody in 9 out of 13 patients with lupus nephritis (V)	Strong positive IgG2 anti-AENO antibody in lupus nephritis (II, III, IV, V)

Regard no detection of α-enolase in glomeruli from primary MN in our study, we used human IgG-adsorbed primary and secondary antibodies tested on frozen sections gave no signal of α-enolase in glomeruli but still well visible staining in proximal tubules. These discrepancies between the two studies should be investigated further in a future study.

We agree with Dr. Murtas’s comments that the combination of antigenic α-enolase epitopes and IgG subclasses may differ by disease. We have to pay attention to anti-AENO IgG subclasses, which may shed new light on the mechanism of MN and other diseases.

## References

[1] Kimura Y, Miura N, Debiec H, Morita H, Yamada H, Banno S, Ronco P, Imai H (2016). Circulating antibodies to alpha-enolase and phospholipase A_2_ receptor and composition of glomerular deposits in Japanese patients with primary or secondary membranous nephropathy. Clin Exp Nephrol..

[2] Murtas C, Bruschi M, Candiano G, Moroni G, Magistroni R, Magnano A, Bruno F, Radice A, Furci L, Argentiero L, Carnevali ML, Messa P, Scolari F, Sinico RA, Gesualdo L, Fervenza FC, Allegri L, Ravani P, Ghiggeri GM (2012). Coexistence of different circulating anti-podocyte antibodies in membranous nephropathy. Clin J Am Soc Nephrol.

[3] Bruschi M, Sinico RA, Moroni G, Pratesi F, Migliorini P, Galetti M, Murtas C, Tincani A, Madaio M, Radice A, Franceschini F, Trezzi B, Bianchi L, Giallongo A, Gatti R, Tardanico R, Scaloni A, D’Ambrosio C, Carnevali ML, Messa P, Ravani P, Barbano G, Bianco B, Bonanni A, Scolari F, Martini A, Candiano G, Allegri L, Ghiggeri GM (2014). Glomerular autoimmune multicomponents of human lupus nephritis in vivo: alpha-enolase and annexin AI. J Am Soc Nephrol.

